# Supplementary material for: Accelerating AutoDock Vina with GPUs
Source: Molecules. 2022 May 9;27(9):3041. doi: 10.3390/molecules27093041 (PMC9103882; doi:10.3390/molecules27093041)
Supplement: Supplementary file 1 [file molecules-27-03041-s001.zip › Vina_GPU_SI.pdf]

Accelerating AutoDock Vina with GPUs

Shidi Tang,[†,‡] Ruiqi Chen,[¶] Mengru Lin,[¶] Qingde Lin,[§] Yanxiang Zhu,[¶] Ji Ding,[†,‡] Haifeng Hu,[‖] Ming Ling,[§] and Jiansheng Wu*,[†,‡]

†*School of Geographic and Biological Information, Nanjing University of Posts and Telecommunications, Nanjing 210023, China*

‡*Smart Health Big Data Analysis and Location Services Engineering Research Center of Jiangsu Province, Nanjing 210023, China*

¶*VeriMake Research, Nanjing Renmian Integrated Circuit Technology Co., Ltd., Nanjing 210088, China*

§*National ASIC System Engineering Technology Research Center, Southeast University, Nanjing 210096, China*

‖*School of Telecommunication and Information Engineering, Nanjing University of Posts and Telecommunications, Nanjing 210003, China*

E-mail: jansen@njupt.edu.cn

Supplementary Information

Supplementary Tables

Table S1: An example of config.txt file

receptor = ./2bm2_protein.pdbqt
ligand = ./2bm2_ligand.pdbqt
center_x = 40.415
center_y = 110.986
center_z = 82.673
size_x = 24.0
size_y = 26.25
size_z = 22.5

Table S2: Descriptions of experimental data

PDBid	N_{atom}	N_{rot}	PDBid	N_{atom}	N_{rot}	PDBid	N_{atom}	N_{rot}	PDBid	N_{atom}	N_{rot}
5tim	5	0	1j3j	22	3	1jla	28	7	2x97	39	13
1aet	6	0	1p62	22	4	1r58	28	10	1os0	39	13
1ac8	8	0	1tz8	22	6	1t40	29	6	4tmn	39	14
2j5s	11	2	1u1c	22	7	1yvf	29	6	1t46	40	6
1w1p	12	0	1mmv	22	8	1unl	29	7	3bkk	40	14
1n2j	12	5	1u4d	23	0	1xoz	30	1	3ov1	41	12
1p2y	13	1	1sqn	23	2	1l7f	30	8	2xhm	42	14
2rdr	13	5	1yv3	23	2	1vcj	30	10	1h23	42	15
1lrh	14	2	4fev	23	3	1opk	31	4	4djr	43	15
1tni	14	5	2brt	23	4	1m2z	31	5	7cpa	43	15
1jd0	15	2	1k3u	23	6	2br1	31	7	3l3n	43	16
1n1m	15	3	1q41	24	1	2bsm	31	7	1w3l	43	16
1uwc	15	4	1t9b	24	3	1z95	31	8	2r23	43	19
1hww	16	3	1ia1	24	3	1qi0	31	12	3s8o	44	12
1q4g	17	3	1hp0	24	5	1a30	31	13	5kao	44	15
1tt1	17	4	1nav	24	6	3l4u	31	17	1sln	44	17
1of6	17	5	1meh	24	7	1s3v	32	6	2d1o	44	23
1x8x	17	5	1gkc	24	8	1hwi	32	10	1kzk	45	11
1hnn	18	2	3kwa	24	13	2bm2	33	7	3zso	45	13
1r9o	18	3	1gm8	25	4	1s19	33	8	5wlo	46	10
1n2v	18	3	1yqy	25	5	1v0p	33	8	1ygc	47	13
1sq5	18	8	1v48	25	6	1r1h	33	10	1u33	47	18
4elm2	18	14	1jje	25	7	1oyt	34	4	4gid	53	17
2ifb	18	14	1v4s	26	4	1hvy	34	9	3utu	54	16

1uou	19	2	1ke5	26	4	1pmn	35	6	1hfs	54	18
1hq2	19	2	1q1g	26	5	1sj0	36	8	2xy9	54	18
1tow	19	4	1g9v	26	6	3oe5	36	11	3pww	55	14
1l2s	20	4	1lbk	26	11	1lpz	37	6	1u1b	55	16
1w2g	20	4	2wbg	26	11	1y6b	37	8	3cyx	56	14
1sg0	20	5	1owe	27	3	1uml	37	11	1jyq	60	20
1xm6	20	5	1oq5	27	5	3bpc	37	15	3drf	63	26
3uex	20	16	3tmn	27	7	1ywr	38	5	2vaa	87	32
1of1	21	4	1xoq	27	7	1mzc	38	8	2er7	89	32
1ig3	21	6	1r55	27	9	10gs	38	14	4er4	93	30
1gpk	22	1	1n46	28	5	3coy	39	11	3er5	108	31

N_{atom}: number of atoms in a ligand molecule.

N_{rot}: number of totatable bonds in a ligand molecule.

Table S3: Details of GPU cards used in this work

Device	Peak Compute Performance		CUDA cores	On-board memory	Architecture
	FP32	FP64			
NVIDIA Geforce GTX 1080ti	11.34 TFLOPS	354.4 GFLOPS	3584	6GB	Pascal
NVIDIA Geforce RTX 2080Ti	13.45 TFLOPS	420.2 GFLOPS	4352	11GB	Turing
NVIDIA Geforce RTX 3090	35.58 TFLOPS	556.0 GFLOPS	10496	24GB	Ampere

Both NVIDIA Geforce GTX 1080ti and NVIDIA Geforce RTX 2080ti are carried with Intel(R) Xeon(R) CPU E5-2678 v3 @ 2.50GHz; NVIDIA Geforce RTX 3090 is carried with Intel (R) Core (TM) i9-10900K CPU @ 3.7 GHz.

Supplementary Figures

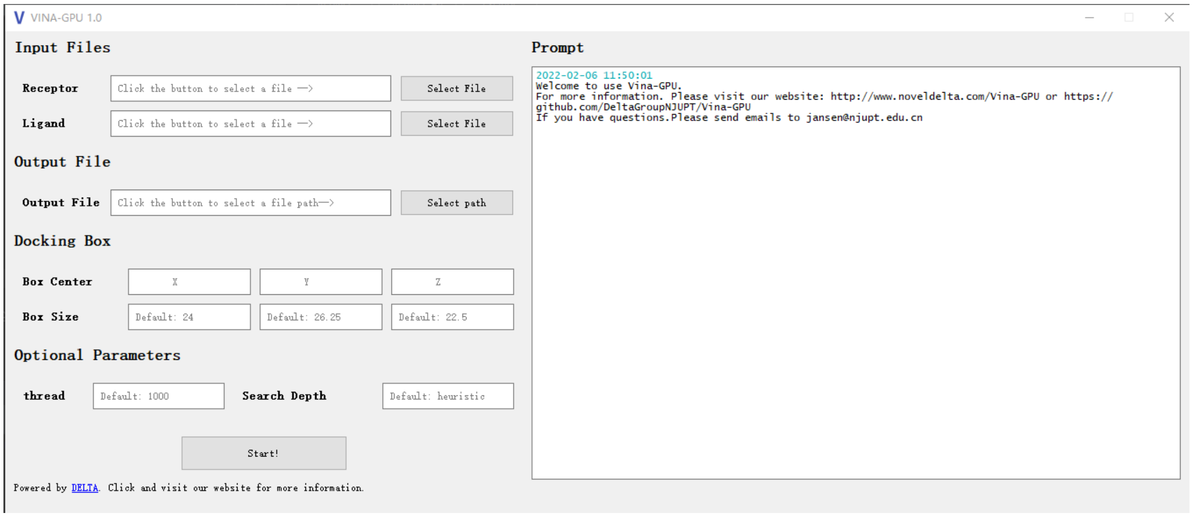

Figure S1: Graphic user interface (GUI) of Vina-GPU. It consists of five main components (Figure S9). The first one is the *input files* that defines both the ligand and the receptor, these files can be easily chosen by a *select file* button. The second one is the *output files* that determine the final output file of the ligand. The third one is the *docking box* that includes *box center* and *box size*, which determine the position and the size of a docking pocket. The fourth one is the configuration of optional values for parameters including *thread* and *search_depth*. The last part is the running windows for outputting relevant execution information. Finally, Vina-GPU can be executed by the *start* button.

Supplementary Text

Text S1. Guideline for running Vina-GPU (see http://www.noveldelta.com/Vina_GPU or https://github.com/DeltaGroupNJUPT/Vina-GPU)
